# What Is the Future of Primary Mental Health Care?: A Post COVID-19 Service Evaluation

**DOI:** 10.1192/bjo.2022.412

**Published:** 2022-06-20

**Authors:** Ahmad Zarif, Imaduldin Nazir, Azad Mahmod, Hajira Bibi, Reshma Rasheed, Anjali Patel, Yathorshan Shanthakumaran

**Affiliations:** 1 New Vision University, Tbilisi, Georgia; 2 Rigg Milner Medical Centre, East Tilbury, United Kingdom

## Abstract

**Aims:**

During the COVID-19 pandemic, many service lines needed to be transformed to enable more telemedicine and virtual consultations. This enabled seamless care across many service boundaries as all services adapted to operate virtually. During COVID-19, the mental health of many patients deteriorated. With easing of restrictions, we wanted the patient voice to be heard and to ensure our service was patient-centred. We undertook a service evaluation to understand our patients preferences. Our cross-sectional study evaluated patient preferences for their care which we felt was important as earlier during pandemic, patients did not have the choice to choose between virtual vs face-to-face consultations. We felt this was important to our patients so they could exercise choice of consultation and this would enable the patient voice to be heard.

**Methods:**

591 patients across three practices in primary care were identified from the Serious Mental Illness (SMI) and on the depression register. They were asked about their preference of care: telemedicine vs face-to-face consultations. Using a simple questionnaire, in order to record their preference on the patient screen. Of these a total of 495 patients (83%) participated in the study.

**Results:**

Of the 495 respondents, 308 (52%) declined virtual telemedicine consultations and 175 (29%) patients were content with virtual consultations. Of the 175 patients who wanted telemedicine were 20 to 40 years of age. Reasons given included convenience (allows family and work commitment) and overall time management (reluctancy to travel). The 308 patients (52%) wanted face-to-face consultations because they wanted human contact, validation of their mental health problems, reassurance and were uncomfortable about discussions on the phone. They also had poor mobility especially the elderly who chose traditional models of care.

**Conclusion:**

As services are restored to the new norm of patient care, patient choice should remain paramount if services are to remain patient centric. During the COVID-19 pandemic, many services transformed to virtual consultation of necessity without recognising the impact on patients themselves. Patients with serious mental health and depression are inherently vulnerable and our evaluation goes to show that despite the popularity of telemedicine. Patient choice should enable patients to access face-to-face care for greater patient satisfaction.

